# A Comprehensive Analysis of HOXB13 Expression in Hepatocellular Carcinoma

**DOI:** 10.3390/medicina60050716

**Published:** 2024-04-26

**Authors:** Eun-A Jeong, Moo-Hyun Lee, An-Na Bae, Jongwan Kim, Jong-Ho Park, Jae-Ho Lee

**Affiliations:** 1 Department of Anatomy, Keimyung University School of Medicine, Daegu 42601, Republic of Korea; wjddmsdk2706@naver.com (E.-A.J.); sand31@hanmail.net (A.-N.B.); jpark@dsmc.or.kr (J.-H.P.); 2 Department of Surgery, Keimyung University School of Medicine, Daegu 42601, Republic of Korea; mhlee197@dsmc.or.kr; 3 Department of Biomedical Laboratory Science, Dong-Eui Institute of Technology, 54 Yangji-ro, Busan 47230, Republic of Korea; dahyun@dit.ac.kr

**Keywords:** hepatocellular carcinoma, HOXB13, MMP9, E2F1, MEIS1, big data

## Abstract

*Background and objectives*: Hepatocellular carcinoma (HCC) is one of the most common malignancies worldwide and is caused by multiple factors. To explore novel targets for HCC treatment, we comprehensively analyzed the expression of HomeoboxB13 (HOXB13) and its role in HCC. *Materials and Methods*: The clinical significance of HCC was investigated using open gene expression databases, such as TIMER, UALCAN, KM, OSlihc, and LinkedOmics, and immunohistochemistry analysis. We also analyzed cell invasion and migration in HCC cell lines transfected with HOXB13-siRNA and their association with MMP9, E2F1, and MEIS1. *Results*: HOXB13 expression was higher in fibrolamellar carcinoma than in other histological subtypes. Its expression was associated with lymph node metastasis, histological stage, and tumor grade. It was positively correlated with immune cell infiltration of B cells (R = 0.246), macrophages (R = 0.182), myeloid dendritic cells (R = 0.247), neutrophils (R = 0.117), and CD4+ T cells (R = 0.258) and negatively correlated with immune cell infiltration of CD8+ T cells (R = −0.107). A positive correlation was observed between HOXB13, MMP9 (R = 0.176), E2F1 (R = 0.241), and MEIS1 (R = 0.189) expression (*p* < 0.001). The expression level of HOXB13 was significantly downregulated in both HepG2 and PLC/PFR/5 cell lines transfected with HOXB13-siRNA compared to that in cells transfected with NC siRNA (*p* < 0.05). Additionally, HOXB13 significantly affected cell viability and wound healing. *Conclusions*: HOXB13 overexpression may lead to poor prognosis in patients with HCC. Additional in vivo studies are required to improve our understanding of the biological role and the exact mechanism of action of HOXB13 in HCC.

## 1. Introduction

Liver hepatocellular carcinoma (LIHC) is a common malignancy worldwide. LIHC is a cancer with a poor prognosis and high mortality rate [1]. Hepatocellular carcinoma (HCC) is the most common type of LIHC, accounting for approximately 90% of all primary liver cancers, whereas fibrolamellar carcinoma (FLC) and hepatoblastoma are rare types [2]. HCC has a poor prognosis and a high mortality rate [3]. Although clinical treatment of HCC has recently made significant progress, patients with HCC succumb to death within 6–20 months and the 5-year survival rate is only 18% [4]. HCC occurs most often in association with chronic liver inflammation and cirrhosis. The treatment methods for HCC vary greatly depending on the stage of the disease and the patient’s condition [5]. The representative treatment methods include surgical resection and liver transplantation [6]. Surgery is possible in less than 40% of cases, and the recurrence rate after surgery is high [7]. As multiple factors are involved in HCC pathogenesis, exploring the molecular mechanisms underlying HCC treatment is clinically important.

HomeoboxB13 (HOXB13) is a sequence-specific transcription factor that preferentially binds methylated DNA and encodes a transcriptional regulator of cell growth and differentiation during embryonic development [8]. HOXB13 is expressed in the developing urogenital tract and plays diverse roles in carcinogenesis and inflammatory diseases. Previous studies have suggested that HOXB13 functions as a tumor suppressor in colon and kidney cancers and that inactivation of HOXB13 may play an important role in tumor formation and metastasis [9,10]. However, it has been found to be overexpressed in several tumors, such as breast, ovarian, and cervical cancers [11,12,13]. These results demonstrate that HOXB13 plays different roles in cancer progression. A recent study identified strong localization of HOXB13 in patients with liver fibrosis, suggesting an important role for HOXB13 in the pathogenesis of liver fibrosis [14]. As liver fibrosis is a major risk factor for HCC, it is important to understand the role and molecular functions of HOXB13 in HCC [15]. However, currently, there is limited information about the clinicopathological characteristics and prognosis of HOXB13 in HCC, and its clinical significance remains unclear.

In this study, clinical and prognostic values of LIHC were analyzed through bioinformatics big data analysis based on the Total Cancer Genome Atlas (TCGA). We analyzed the association between related genes such as MMP9, E2F1, and MEIS1 in HCC cell lines transfected with HOXB13-siRNA and examined the role of HOXB13 in cell viability and migration. The results of this study provide a foundation for demonstrating its potential as a novel biomarker for the treatment of HCC.

## 2. Materials and Methods

### 2.1. TIMER Database Analysis

TIMER is a TCGA database used to analyze immune infiltration and clinical data (age, sex, race, and stage) of various cancer types and specific genes. We analyzed the relationship between HOXB13 expression and survival rate, immune cell infiltration, and cancer prognosis in LIHC through correlation analysis between immune cells. In addition, we analyzed the role of HOXB13 in LIHC and its correlation with immune-infiltrating cells, including B cells, macrophages, myeloid dendritic cells, neutrophils, CD4+ T cells, and CD8+ T cells. 

### 2.2. UALCAN Database Analysis

The UALCAN database (http://ualcan.path.uab.edu (accessed on 17 January 2024)) is a web resource for cancer data analysis that uses clinical data from TCGA Level 3 RNA-seq and LIHC. We compared the relative expression of HOXB13 according to sex, age, other clinicopathological characteristics, lymph node metastasis, cancer stage, and tumor grade.

### 2.3. Immunohistochemistry (IHC) Staining Analysis

The Human Proteome Atlas (HPA) database (https://www.proteinatlas.org/ (accessed on 17 January 2024)) contains the largest and most comprehensive information on protein distribution in human tissues and cells and explores single-cell expression patterns. To analyze the HOXB13 proteomic expression levels, we obtained IHC images of HOXB13 proteomic expression from the HPA for LIHC. The proteomic expression level of HOXB13 was graded as undetectable or medium based on the intensity of staining and the fraction of stained cells.

### 2.4. KM Database Analysis

KM (https://kmplot.com/analysis/ (accessed on 17 January 2024)) is based on an online database and includes survival rates, such as overall survival (OS), relapse-free survival (RFS), and clinical data of patients with LIHC. Survival data for HOXB13 were analyzed using the log-rank test.

### 2.5. OSlihc Database Analysis

OSlihc (http://bioinfo.henu.edu.cn/DatabaseList.jsp (accessed on 16 January 2024)) provides a platform for researchers to discover new prognostic biomarkers and provides the opportunity to create novel targeted therapies for LIHC. To clarify the predictive value of genes in OSlihc, survival data, such as OS, disease-specific survival (DSS), and progression-free interval (PFI), were generated; OS was measured in all cohorts and combined cohorts, whereas PFI and DSS were analyzed using TCGA.

### 2.6. LinkedOmics Database Analysis

The LinkedOmics database (http://www.linkedomics.org/admin.php (accessed on 16 January 2024)) is a web-based platform for analyzing 32 TCGA cancer-associated multidimensional datasets. Correlations between HOXB13-associated genes were analyzed using Pearson’s correlation coefficient. Gene Ontology (GO) and Kyoto Encyclopedia of Genes and Genomes (KEGG) pathway enrichment analyses were conducted using a Database for Annotation, Visualization, and Integrated Discovery (DAVID 6.8, v6.8; https://david.ncifcrf.gov/home.jsp (accessed on 17 January 2024), and an online bioinformatics tool (http://www.bioinformatics.com.cn (accessed on 17 January 2024)).

### 2.7. Cell Culture and siRNA Transfection

The human HCC cell lines, HepG2 and PLC/PRF/5, were purchased from the Japanese Collection of Research Bioresources Cell Bank (JCRB Cell Bank, Osaka, Japan) and American type culture collection (ATCC), respectively. HepG2 and PLC/PRF/5 cells were cultured in Dulbecco’s modified Eagle medium supplemented with 1% penicillin/streptomycin solution and 10% fetal bovine serum (Gibco BRL., Grand Island, NY, USA) in a humidified 5% CO_2_ incubator at 37 °C. The target sequence of HOXB13-siRNA was as follows: 5-(GGUCAUGGUUUGUUGAGCA)-3 (Ambion, Cat. 4392421). Lipofectamine (Invitrogen, Waltham, MA, USA) and HOXB13-siRNA were mixed in Opti-MEM (Thermo Scientific, Rockford, IL, USA). After 48 h of HOXB13-siRNA transfection, RNA was extracted. Total cellular RNA was extracted using the QIAzol lysis reagent (Qiagen, Redwood City, CA, USA) according to the manufacturer’s protocol. A NanoDrop ND-1000 spectrophotometer (Thermo Fisher Scientific, Waltham, MA, USA) was used to determine the quantity and quality of isolated total cellular RNA.

### 2.8. Quantitative Real-Time PCR Analysis (RT-qPCR)

Reverse-transcription reactions were conducted using ReverTra Ace qPCR RT Master Mix (TOYOBO, Osaka, Japan). The expression levels of HOXB13, MMP9, E2F1, MEIS1, and GAPDH were measured using RT-qPCR. RT-qPCR was performed using a CFX Connect RT-PCR System (Bio-Rad, Hercules, CA, USA). The primer sequences used for RT-qPCR are listed in Table 1. The PCR amplification cycles were as follows: 95 °C for 10 min, followed by 40 cycles of 95 °C for 60 s and 72 °C for 30 s. 

### 2.9. Cell Viability Assay

HepG2 and PLC/PRF/5 cells were seeded in 24-well plates at a density of 2 × 10^5^ cells/well. After 24 h, 48 h, and 72 h after siRNA transfection at 37 °C, the cells were subsequently incubated with 100 μL of 5 mg/mL MTT for 4 h. Cell viability was subsequently analyzed at a wavelength of 570 nm using an Asys UVM 340 microplate reader (Biochrom, Cambridge, UK). Each experiment was performed in triplicate.

### 2.10. Wound Healing Assay

HepG2 and PLC/PRF/5 cells (2 × 10^5^) were seeded in 12-well plates at 70–80% confluence for the wound healing assay. After washing, the cells were transfected with negative control siRNA or HOXB13-siRNA using Lipofectamine 2000 (Invitrogen, Camarillo, CA, USA) according to the manufacturer’s instructions. After 6 h of transfection, the medium was replaced with standard culture medium. The cells were incubated at 37 °C for 24 h, 48 h, and 72 h. Light microscope images of three locations of the marked wounds were obtained, and the migrated cells were counted.

### 2.11. Statistical Analysis

We used the TIMER and UALCAN databases to analyze the gene expression data. We employed online tools, such as the KM plotter, TIMER, and OSlihc, to generate the KM curves. Survival outcomes are presented as HRs with *p*-values from the log-rank test. The TIMER database was used to assess the correlation between the gene expression levels and immune signature scores using Spearman’s correlation coefficients. All data were sourced from publicly accessible databases and analyses were conducted using online tools. The Mann–Whitney U test was used to test the significance of mRNA expression levels and other quantitative measurements when comparing the two groups. All reported results include *p*-values derived from the log-rank test. Statistical significance was set at *p* < 0.05.

## 3. Results

### 3.1. Assessment of HOXB13 Expression in Different Cancer and Normal Tissues

**Figure 1:**
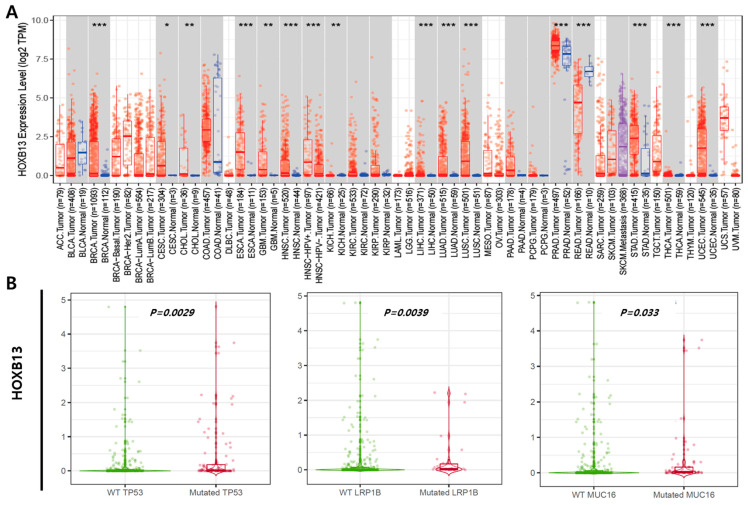
Expression level of HOXB13 observed through database. (**A**) Expression levels of normal and tumor tissues of HOXB13 in various types of cancer. (**B**) Comparison of HOXB13 expression in the TP53, LRP1B, and MUC16 mutants vs. WT LIHC groups via the TIMER 2.0 database. * *p*  <  0.05; ** *p*  <  0.01; *** *p*  <  0.001.

We used TIMER to examine RNA sequencing data from the TCGA and evaluated HOXB13 expression levels in specific tumor types. HOXB13 expression between normal and tumor tissues significantly differed in breast invasive carcinoma (*p* < 0.001), cholangiocarcinoma (*p* < 0.01), esophageal carcinoma (*p* < 0.001), head and neck squamous cell carcinoma (*p* < 0.001), head and neck squamous cell carcinoma HPV (*p* < 0.001), liver hepatocellular carcinoma (*p* < 0.001), lung adenocarcinoma (*p* < 0.001), lung squamous cell carcinoma (*p* < 0.001), prostate adenocarcinoma (*p* < 0.001), rectum adenocarcinoma (*p* < 0.001), stomach adenocarcinoma (*p* < 0.001), thyroid carcinoma (*p* < 0.001), and uterine corpus endometrial carcinoma (*p* < 0.001) (Figure 1A). Gene mutations exist depending on the cancer type, and HOXB13 expression in LIHC differed significantly depending on mutation status. HOXB13 expression in LIHC patients with TP53 (1.179 fold), LRP1B (0.357 fold), and MUC16 (0.468 fold) mutations was statistically higher than that in the wild type (*p* < 0.05) (Figure 1B). 

### 3.2. Prognostic Value of HOXB13 mRNA Expression in LIHC

**Figure 2:**
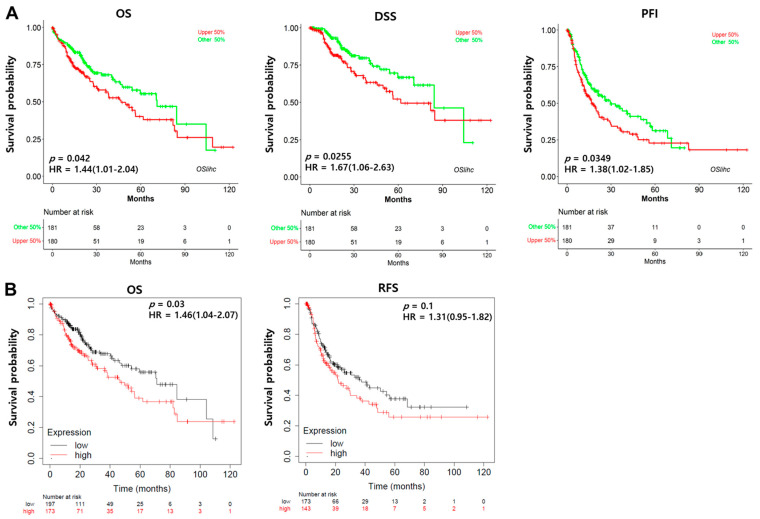
Prognostic significance of high expression of HOXB13 in LIHC. (**A**) Evaluation of the prognostic value of HOXB13 in Oslihc. *p*-value, confidence interval (95% CI) and number at risk are as shown. The *y*-axis represents survival rate, while the *x*-axis represents survival time (months). (**B**) The prognostic value of HOXB13 was analyzed using KM.

We used the OSlihc database to identify HOXB13 as a novel candidate biomarker and confirmed its significant association with survival. Patients with high HOXB13 expression in LIHC had a shorter OS (HR, 1.44; *p* < 0.05) and DSS (HR = 1.67, *p* < 0.05) (Figure 2A). Additionally, the Kaplan–Meier plot was used to determine the prognostic significance of HOXB13 expression in LIHC. High HOXB13 expression showed poor OS (HR = 1.46, *p* < 0.05). However, RFS (HR = 1.31, *p* = 0.1) was not statistically significant (Figure 2B).

### 3.3. Clinical Characteristics of HOXB13 Expression in LIHC using the UALCAN Database 

**Figure 3:**
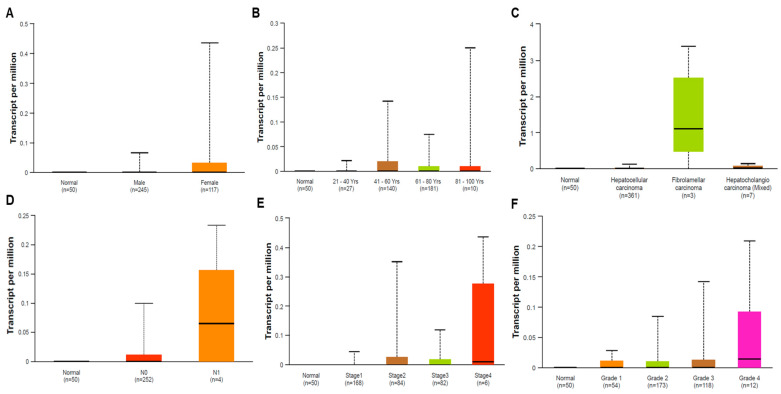
Relative HOXB13 expression in normal and different subgroups of LIHC. (**A**) Sex. (**B**) Age. (**C**) Histological subtypes. (**D**) Nodular metastasis. (**E**) Stage. (**F**) Grade.

The clinicopathological characteristics of HOXB13 expression in LIHC are shown in Figure 3. HOXB13 showed higher expression in females than in males, and it was more highly expressed in the ages of 41–60 years (Figure 3A,B). HOXB13 was more highly expressed in FLC than in the other histological subtypes (Figure 3C). Additionally, HOXB13 expression was associated with lymph node metastasis, histological stage, and tumor grade (Figure 3D–F).

### 3.4. Association of HOXB13 Expression with Immune Cell Infiltration in LIHC

**Figure 4:**
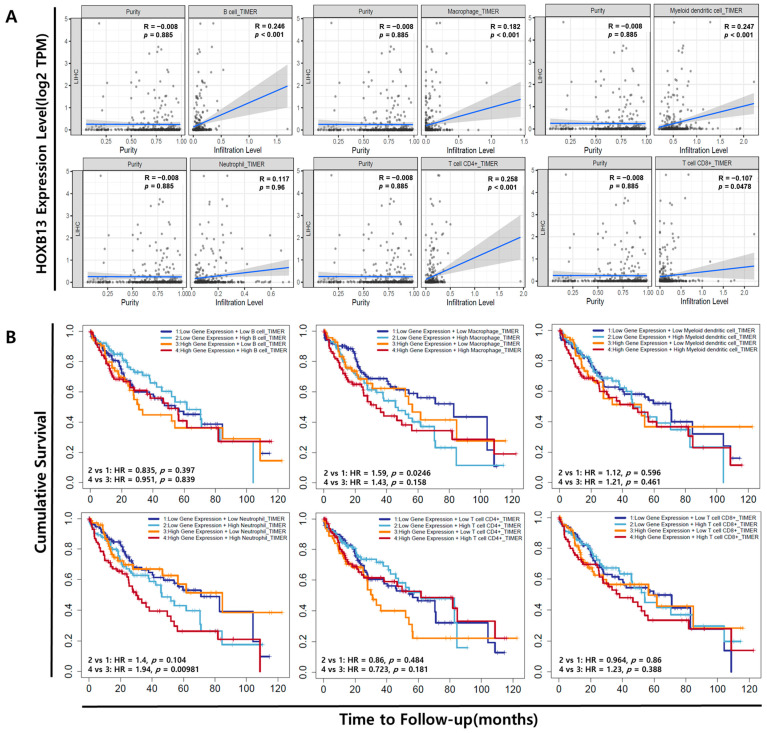
Correlation between HOXB13 and infiltrated immune cells in LIHC. (**A**) Correlation analysis between HOXB13 and infiltrated immune cells using the TIMER database. (**B**) Survival analysis according to HOXB13 gene expression and expression of infiltrated immune cells observed in TIMER database.

We investigated the correlation between HOXB13 and six tumor immune-infiltrating cells using the TIMER database. Immune cell infiltration generally accelerates cancer progression and affects survival outcomes. TIMER is a tool for analyzing the association of specific genes with immune infiltration. HOXB13 expression was positively correlated to B cells (R = 0.246, *p* < 0.001), macrophages (R = 0.182, *p* < 0.001), myeloid dendritic cells (R = 0.247, *p* < 0.001), neutrophils (R = 0.117, *p* = 0.96), CD4+ T cells (R = 0.258, *p* < 0.001), and CD8+ T cells (R = −0.107, *p* = 0.0478) (Figure 4A). The correlation between HOXB13 and LIHC expression, abundance of immune infiltrates (B cells, macrophages, myeloid dendritic cells, neutrophils, and CD4+ and CD8+ T cells), and survival time are shown in Figure 4B. Patients with low HOXB13 gene expression and low macrophage infiltration had longer survival times than those with low gene expression and high macrophage infiltration (*p* < 0.05). Patients with high HOXB13 gene expression and low neutrophil infiltration had longer survival times than those with high gene expression and high neutrophil infiltration (*p* < 0.01). B cells, myeloid dendritic cells, and CD4+ and CD8 + T cells did not have a prognostic value. Thus, cumulative curve analysis showed that immune infiltrates were significantly associated with HOXB13 in LIHC, indicating that immune-infiltrating cells significantly affected prognosis. In this study, we found a significant correlation between survival time and the HOXB13 expression in the infiltration of two immune cell types, macrophages and neutrophils, indicating that HOXB13 expression in combination with immune cell status can predict prognosis.

### 3.5. Protein Expression of HOXB13 in LIHC

**Figure 5:**
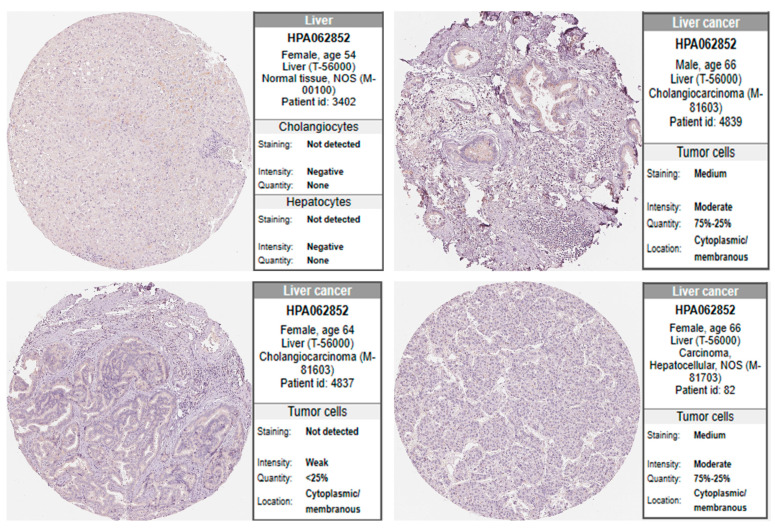
IHC analysis of protein expression level of HOXB13 in LIHC. Protein expression of HOXB13 was analyzed using the Human Protein Atlas (HPA) database.

The protein expression levels of HOXB13 were obtained from the HPA database. IHC images of the HPA revealed that the protein expression of HOXB13 was undetected in normal liver tissue, whereas protein expression of HOXB13 was significantly higher in LIHC tissues (Figure 5). Our results revealed the overexpression of HOXB13 at the transcriptional and translational levels in patients with LIHC.

### 3.6. Co-Expression Analysis of the HOXB13 Gene

**Figure 6:**
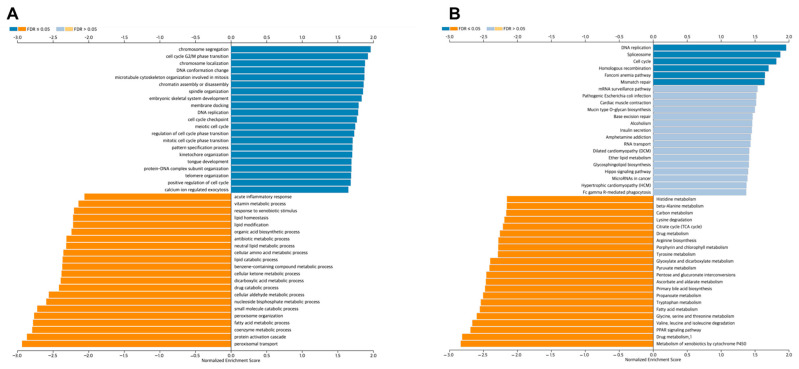
Co-expression analysis of HOXB13 in LIHC according to LinkedOmics database. (**A**) Correlation between HOXB13 expression and GO biological processes. (**B**) Correlation between HOXB13 expression and KEGG pathway.

GO (biological processes, cellular components, and molecular functions) and KEGG pathway analyses of HOXB13-related genes were performed to explore the molecular mechanism of HOBX13 in HCC regulation. GO annotations presented that HOXB13 co-expressed genes were predominantly co-expressed during chromosome segregation, cell cycle G2/M phase transition, chromosome localization, DNA conformational changes, microtubule cytoskeleton organization involved in mitosis, chromatin assembly or disassembly, etc. (Figure 6A). KEGG pathway assays demonstrated the enrichment of DNA replication, spliceosomes, the cell cycle, homologous recombination, the Fanconi anemia pathway, and mismatch repair (Figure 6B). These data suggest that HOXB13 may be associated with DNA replication, cell cycle, and proliferation. 

**Figure 7:**
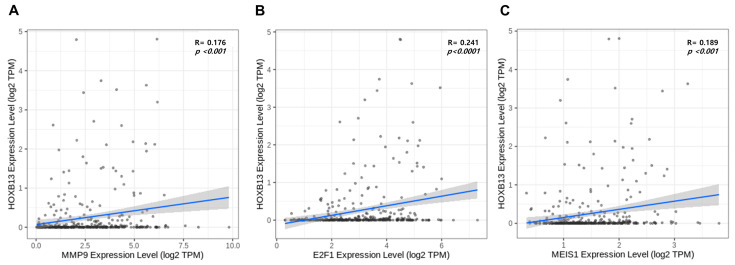
Correlation analysis between (**A**) HOXB13 expression and MMP9. (**B**) HOXB13 expression and E2F1. (**C**) HOXB13 expression and MEIS1.

Therefore, we examined the expression level of MMP9, E2F1, and MEIS1 based on the above result. Then, the correlation between the expression levels of HOXB13, MMP9, E2F1, and MEIS1 was determined. The gene–gene correlation analysis results indicated that HOXB13 expression was positively correlated with the expression of MMP9 (R = 0.176, *p* < 0.001), E2F1 (R = 0.241, *p* < 0.0001), and MEIS1 (R = 0.189, *p* < 0.001) (Figure 7A–C).

### 3.7. Effect of HOXB13 Silencing on MMP9, E2F1, and MEIS1 mRNA Expression

**Figure 8:**
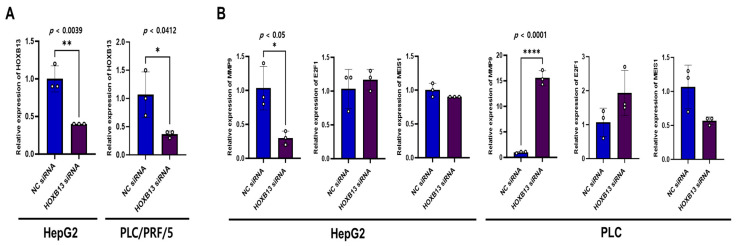
siRNA knockdown of HOXB13 in HCC cells and the association between MMP9, E2F1, MEIS1 mRNA expression. (**A**) Detection of HOXB13 mRNA expression in HepG2, PLC/PRF/5 cells at 48 h after transfection. (**B**) MMP9, E2F1, and MEIS1 mRNA expression in HepG2 and PLC/PRF/5 after HOXB13 knockdown. * *p* < 0.05, ** *p* < 0.01, **** *p* < 0.0001.

To confirm these results, a cell-line assay was performed. We explored the association between HOXB13 and MMP9, E2F1, and MEIS1 as cell cycle- and proliferation-associated genes in HOXB13-knocked HepG2 and PLC/PRF/5 cells. HOXB13 was knocked down in HepG2 and PLC/PRF/5 cells, and cells transfected with the negative control siRNA were used as controls. To confirm the relationship between HOXB13, MMP9, E2F1, and MEIS1, HOXB13-siRNA efficacy was examined using qPCR. As shown in Figure 8A, HOXB13 mRNA levels were significantly lower in both HepG2 and PLC/PRF/5 cells transfected with HOXB13-siRNA than in cells transfected with NC siRNA (*p* < 0.05). Additionally, HOXB13 knockdown significantly reduced the mRNA levels of MMP9 in HepG2 cells (*p* < 0.05); however, it upregulated MMP9 expression in PLC/PRF/5 cells (*p* < 0.0001) (Figure 8B). In PLC/PRF/5 cells transfected with HOXB13-siRNA, MEIS1 expression tended to decrease; however, the difference was not statistically significant (*p* = 0.0569). The other variables did not change significantly.

### 3.8. HOXB13 Knockdown Reduced HCC Cell Viability and Cell Recovery

**Figure 9:**
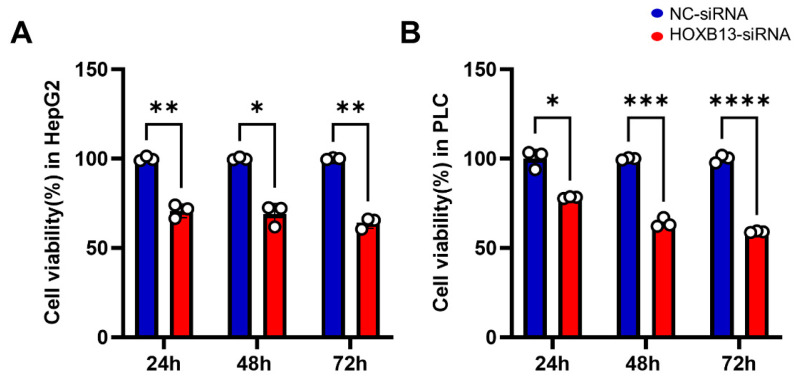
siRNA knockdown of HOXB13 and growth of HCC cells. (**A**) Detection of cell viability after transfection in HepG2. (**B**) Detection of cell viability after transfection in PLC/PRF/5. * *p* < 0.05, ** *p* < 0.01, *** *p* < 0.001, and **** *p* < 0.0001.

**Figure 10:**
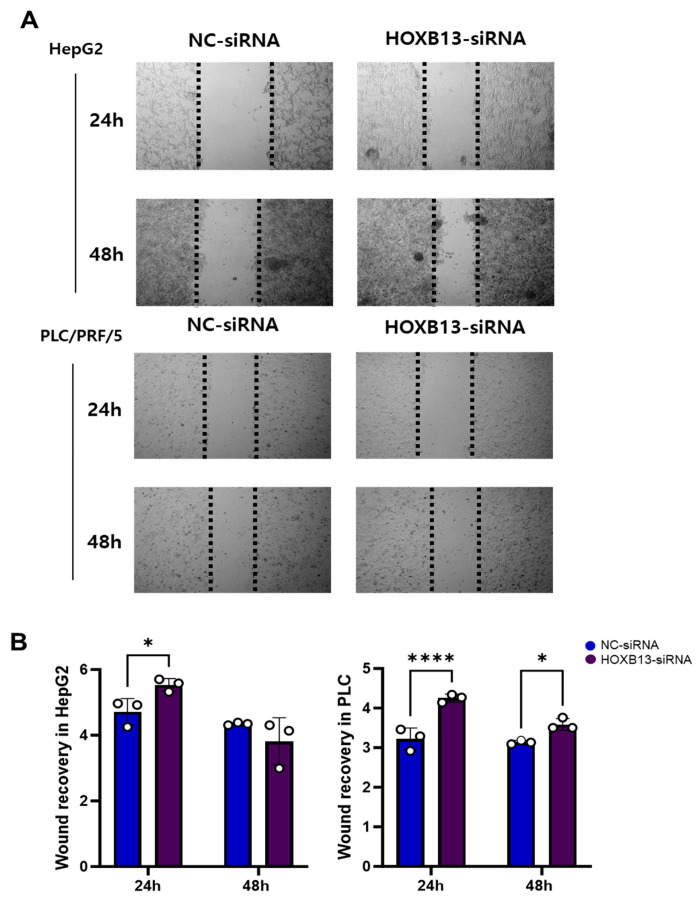
Cell migration in cell scratch wound healing assay. HOXB13 inhibited cell migration in HepG2, PLC/PRF/5. (**A**) Wound healing assay of HepG2 and PLC/PRF/5 for 24 h and 48 h. The HCCs treated with NC-siRNA were used as control. (**B**) Quantification of wound area in control and HOXB13-treated HepG2, PLC/PRF/5. * *p* < 0.05, **** *p* < 0.0001.

The viabilities of HepG2 and PLC/PRF/5 cells were analyzed using the MTT assay. A statistically significant decrease was observed in cell viability on analyzing HepG2 and PLC/PRF/5 cell viability 24, 48, and 72 h after HOXB13 knockdown (Figure 9). Additionally, cell recovery was assessed using cell scratch wound healing analysis. Cell elasticity was observed 24 h after transfection with NC-siRNA and HOXB13-siRNA, and the areas of both HepG2 and PLC/PRF/5 cells were significantly wider after HOXB13-siRNA transfection than after NC-siRNA transfection (*p* < 0.05, *p* < 0.0001). After 48 h, the area was wider in PLC/PRF/5 cells (*p* < 0.05); however, there was no difference in HepG2 cells (Figure 10A,B).

## 4. Discussion

In this study, we analyzed open-access big data, such as TIMER, UALCAN, HPA, KM, LinkedOmics, and OSlihc, to confirm the clinical characteristics of HOXB13 in LIHC. HOXB13 function was investigated in HCC cell lines, HepG2 and PLC/PRF/5.

As a result of looking at HOXB13 expression in LIHC through big data, we showed that HOXB13 expression at both the mRNA and protein levels was higher in LIHC tissues than in non-cancerous tissues. Interestingly, its expression level was associated with gene mutations, and this association was frequently found in cancers. Genetic mutations occur in a variety of human cancer types, and TP53, LRP1B, and MUC16 are among the most frequently altered genes [16]. Using TIMER, we observed the association of HOXB13 with data from the top 15 genes with high mutation frequency in LIHC [17]. As a result, statistical significance was confirmed in TP53, LRP1B, and MUC16 genes (*p* < 0.05). TP53 and LRP1B act as tumor suppressor genes, and mutations can lead to poor prognosis [18,19,20]. Overexpression of MUC16 is associated with poor prognosis in several malignant tumors, and mutations are associated with melanoma, etc. [21,22].

And HOXB13 expression was associated with sex, age, and stage and correlated with variables associated with poor prognosis, such as higher N stage and clinical stage. HCC, the most common type of liver cancer, FLC, and the mixed type were included in the big data. In the present study, HOXB13 was overexpressed in FLC. Recent studies have shown that FLC has a better overall prognosis than other primary liver tumors, such as HCC and intrahepatic cholangiocarcinoma. However, FLC is an aggressive tumor due to its rarity, so despite its generally good prognosis, the overall cure rate is low and postoperative recurrence is common [23]. One limitation of our study is that the effect of HOXB13 overexpression on primary tumors and FLC could not be determined. Additional experiments are expected to be helpful in developing treatments for primary tumors and FLC caused by HOXB13. 

Survival analysis using OSlihc data and a Kaplan–Meier plotter showed that HOXB13 expression was associated with a poor prognosis. All prognostic results, including OS, DSS, PFI, and RFS, were similar, indicating that HOXB13 may act as an oncogene in LIHC. Previous studies demonstrated HOXB13 as a tumor suppressor in colon and kidney cancer [9,10]. However, it and our data suggested that HOXB13 plays various roles depending on the cancer type. Additional research is needed to determine what role HOXB13 plays depending on the type of cancer.

Previous studies have shown that inflammation is essential for driving cancer development through tissue damage and that neutrophils play an essential role in this process. Additionally, the results show that neutrophils are strongly associated with cancer growth promotion and metastasis [24]. Therefore, we investigated the level of immune infiltration of HOXB13 in LIHC. Reverse permeabilization analysis revealed that HOXB13 expression was significantly associated with various immune cell types in LIHC. Analysis using the TIMER database showed that HOXB13 was positively correlated with the infiltration status of immune cells, including macrophages, CD4+ T, CD8+ T, B, and dendritic cells. Although the correlation coefficient was approximately 0.2, all tumor-infiltrating immune cells, except neutrophils, were closely related to tumor formation and development. Interestingly, survival analysis showed that high HOXB13 expression and high neutrophil counts were associated with a worse prognosis than the other groups. Neutrophils are crucial for HCC pathogenesis. They are also involved in tumorigenesis, local tumor progression, and metastasis [25]. Macrophages also showed a prognostic value for HOXB13 expression. Abnormal regulation of macrophages is associated with HCC [26]; however, other immune cells were not associated with LIHC progression. Additional studies with larger sample sizes are needed to validate these results and comprehend the exact mechanism underlying the role of HOXB13 during immune invasion and cancer progression in LIHC. 

GO and KEGG enrichment analyses were performed to elucidate the function of HOXB13 in LIHC. GO data showed that HOXB13 may be involved in cell proliferation and the cell cycle. Based on the role of HOXB13, we observed an association with MMP9, E2F1, and MEIS1, which are genes that play a role in cell proliferation and carcinogenesis. MMPs are one of four subgroups of matrix proteases, and by secreting MMPs, HCC spreads not only to the liver itself but also to distant organs during the course of the disease. High expression of MMP9 is associated with poor survival in HCC, and higher expression and intensity of MMP9 has been shown to increase recurrence [27,28]. The expression level of E2F1 is upregulated in HCC compared to adjacent non-tumor tissue [29]. E2F1 is a transcription factor involved in the regulation of cellular processes such as cell migration and proliferation and generally terminates liver proliferation by activating target gene transcription. That is, it has both apoptotic and proliferative functions, and the detailed role of E2F1 requires further study [29,30]. Additionally, previous studies have shown that tumors with increased MEIS1 levels are potentially less aggressive and tumors with low MEIS1 levels have a high risk of biochemical recurrence and metastasis [31]. 

Based on this hypothesis, gene–gene correlation analysis was performed using big data and cell-line studies. First, big data showed that the expression of HOXB13 was positively correlated with that of MMP9, E2F1, and MEIS1. And then, HCC cell lines were transfected with HOXB13-siRNA to clarify the results in big data. Interestingly, MMP9 expression showed the opposite results in two HCC cell lines transfected with HOXB13-siRNA. Previous studies have shown different results, including showing that HOXB13 negatively regulates the expression and survival of MMP9 and that the expression of MMP9 is not significantly changed when HOXB13 is overexpressed [8,32]. To explain this difference between cell lines and big data, further studies on HOXB13 expression using patient samples are needed, and its molecular mechanisms should also be studied. 

Afterwards, cell viability was confirmed through MTT assay, and migration ability was analyzed through wound healing analysis. HOXB13 knockdown significantly reduced the viability of HCC cells. These results suggest that HOXB13 affects the viability of HepG2 and PLC/PRF/5 cells. This is in agreement with our results from the big data analysis and previous results [8]. In addition, cell recovery through cell scratch wound healing analysis revealed that the gap between cells transfected with HOXB13-siRNA was wider than that between cells transfected with NC-siRNA. Although there were differences in the statistical results depending on the cell type and time, these results indicate that HOXB13 can inhibit the growth and migration of HepG2 and PLC/PRF/5 cells.

## 5. Conclusions

HOXB13 was overexpressed in HCC tissues and was positively correlated with immune cells. HOXB13 promotes the viability and migration of HCC cells, resulting in poor prognosis. These findings provide evidence of a novel regulatory mechanism involving HOXB13 in HCC and suggest that HOXB13 may serve as a promising therapeutic target for HCC.

## Figures and Tables

**Table 1:** Primer sequences used for RT-qPCR in this study.

Name	Primer (5′ to 3′)
HOXB13	Forward: AGCTCCCGTGCCTTATGGTTA
	Reverse: GGCTGGTAGGTTCCCGGAT
MMP9	Forward: TGTACCGCTATGGTTACACTCG
	Reverse: GGCAGGGACAGTTGCTTCT
E2F1	Forward: AGGCCGCCATCCAGGAAAAG
	Reverse: GGATGCCCTCAACGACGTTG
MEIS1	Forward: CACACTGGCCTTAAAGAGG
	Reverse: GTAGATCGTCGTACCTTTGCG
GAPDH	Forward: GAAAGGTGAAGGTCGGAGTC
	Reverse: GTTGAGGTCAATGAAGGGGTC

## Data Availability

The data presented in this study are available on request from the corresponding author.
